# Work productivity loss from depression: evidence from an employer survey

**DOI:** 10.1186/s12913-014-0597-y

**Published:** 2014-12-18

**Authors:** Kathryn M Rost, Hongdao Meng, Stanley Xu

**Affiliations:** Department of Mental Health Law and Policy, College of Behavioral and Community Studies, University of South Florida, 13301 Bruce B. Downs Boulevard, Tampa, FL 33612 USA; School of Aging Studies, College of Behavioral and Community Studies, University of South Florida, 13301 Bruce B. Downs Boulevard, Tampa, FL 33612 USA; Biostatistics, Institute for Health Research, Kaiser Permanente, 10065 E. Harvard Avenue, Suite 300, Denver, CO 80231 USA; Department of Biostatistics and Informatics, School of Public Health, University of Colorado Denver, Denver, USA

**Keywords:** Return on investment, Work, Productivity, Depression, Health promotion

## Abstract

**Background:**

National working groups identify the need for return on investment research conducted from the purchaser perspective; however, the field has not developed standardized methods for measuring the basic components of return on investment, including costing out the value of work productivity loss due to illness. Recent literature is divided on whether the most commonly used method underestimates or overestimates this loss. The goal of this manuscript is to characterize between and within variation in the cost of work productivity loss from illness estimated by the most commonly used method and its two refinements.

**Methods:**

One senior health benefit specialist from each of 325 companies employing 100+ workers completed a cross-sectional survey describing their company size, industry and policies/practices regarding work loss which allowed the research team to derive the variables needed to estimate work productivity loss from illness using three methods. Compensation estimates were derived by multiplying lost work hours from presenteeism and absenteeism by wage/fringe. Disruption correction adjusted this estimate to account for co-worker disruption, while friction correction accounted for labor substitution. The analysis compared bootstrapped means and medians between and within these three methods.

**Results:**

The average company realized an annual $617 (SD = $75) per capita loss from depression by compensation methods and a $649 (SD = $78) loss by disruption correction, compared to a $316 (SD = $58) loss by friction correction (p < .0001). Agreement across estimates was 0.92 (95% CI 0.90, 0.93).

**Conclusion:**

Although the methods identify similar companies with high costs from lost productivity, friction correction reduces the size of compensation estimates of productivity loss by one half. In analyzing the potential consequences of method selection for the dissemination of interventions to employers, intervention developers are encouraged to include friction methods in their estimate of the economic value of interventions designed to improve absenteeism and presenteeism. Business leaders in industries where labor substitution is common are encouraged to seek friction corrected estimates of return on investment. Health policy analysts are encouraged to target the dissemination of productivity enhancing interventions to employers with high losses rather than all employers.

**Trial registration:**

Clinical trials registration number: NCT01013220.

## Background

Productivity loss has been defined as the value of production foregone because of morbidity or mortality [1]. Panels recommend that studies evaluate intervention impact on productivity loss from the societal perspective [2]. However, companies need estimates from the employer perspective to inform their decision-making because companies potentially gain when productivity loss from employee illness is reduced, producing a possible return on investment for offering health benefits that improve productivity. Although national working groups identify the need for return on investment research conducted from the employer perspective [3,4], the field has not developed standardized methods for measuring the basic components of return on investment, including costing out the value of work productivity loss due to illness. The most widely used approach to evaluate productivity loss from an employer perspective has been derived from human capital theory in which the loss of a healthy day represents the loss of productivity whose value in competitive labor markets equals the money wage and fringe (compensation method) [5-7]. However, the empirical literature remains inconclusive in terms of whether this method under-estimates or over-estimates the productivity losses from the employer perspective. On the one hand, traditional compensation methods may under-estimate productivity loss because they do not account for the role of absenteeism in disrupting normal production. As such, some researchers recommend applying upward adjustment to the loss estimates by empirically-derived multipliers described below [8,9]. On the other hand, traditional compensation methods may over-estimate productivity loss by assuming that loss due to illness is not made up by coworkers or contracted labor. As such, other researchers have recommended applying downward adjustment to the loss estimates by using friction methods [10-12]. It remains unclear to what extent illness-related productivity loss estimates utilizing the compensation approach are sensitive to both types of adjustment.

The goal of this study is to characterize between and within variation in illness-related productivity loss from the employer perspective estimated by three methods discussed above. Because the underlying assumptions about workplace disruption from illness vary considerably across the three methods, the research team hypothesizes minimal correlation in estimates of productivity loss calculated by the compensation method, production disruption adjustment, and friction correction adjustment. We elected to study this question in depression because new interventions demonstrate a potential return on investment to companies [13] by reducing absenteeism and increasing productivity at work [14].

## Methods

### Study design

With assistance from 33 employer associations, the research team recruited one senior health benefit professional (hereafter referred to as a respondent) from public or private companies with 100 or more non-unionized workers. Unionized workers were not included in the study because their health benefits are generally negotiated as part of their labor contract. Blinded respondents were randomized to one of two presentations between April 2009 and May 2011 to learn more about state-of-the-art strategies companies could implement to improve depression in the workplace. Detailed information about the study design including eligibility criteria [15] and sample [16] has been previously published. Three hundred twenty five of 403 potential respondents (80.6%) indicating interest in the study attended the assigned presentation. Key variables analyzed in this paper were collected in a short paper-and-pencil survey taken before respondents learned which strategy their presentation addressed. Respondents who failed to complete this survey were re-contacted after the presentation to collect the needed data. Respondents were instructed to consider only their non-unionized employees when answering each question. The protocol and informed consent that respondents signed were approved by the Institutional Review Boards at Florida State University and the University of South Florida.

### Variable definition

Respondent report on his/her company was used to characterize the 325 employers. The generation of productivity loss estimates across the three methods required a range of constructs not available from the employer or any other single source. We used employer-specific estimates of the necessary constructs when they were available. When they were not, we used industry-specific estimates. When neither employer-specific nor industry-specific estimates were available, we used national estimates. Information on data sources is displayed in Table 1.

**Table 1:** **Measurement of key constructs** ^*^

**Construct**	**Source(s)**	**Measurement level**
Depression prevalence	National Comorbity Study adjusted using methods in Greenberg 1996	Industry
	Bureau of Labor Statistics 2010	
Lost absenteeism hours attributable to depression		
Method 1	American Productivity Audit in Stewart 2003 adjusted by Nicholson 2005	Industry
	Table three, Column 4	
Method 2	American Productivity Audit in Stewart 2003 adjusted by Nicholson 2005	Industry
	Table three, Column 4 and 7	
Method 3	American Productivity Audit in Stewart 2003 adjusted by Nicholson 2005	Employer
	Table Three, Column 4 and Table 2 items in current study	
Lost presenteeism hours attributable to depression		
Method 1	American Productivity Audit in Stewart 2003	National
Method 2	American Productivity Audit in Stewart 2003	National
Method 3	American Productivity Audit in Stewart 2003 adjusted by Table two items in current study	Employer
Wage and fringe	Bureau of Labor Statistics 2010	Industry

^*^Full citations provided in the reference section of the current study.

Prevalence - Respondents estimated the number of full-time workers their company currently employed in the United States, and the Year 2000 Bureau of Labor Statistics (BLS) category that best described their company: (1) agriculture, (2) communication (3) construction (4) finance, (5) government, (6) manufacturing, (7) professional, (8) retail, (9) service, (10) transportation/utilities, (11) wholesale, and (12) other. After data collection, the research team recoded each company’s Year 2000 BLS industry category into one of 14 Year 2010 BLS industry categories. Year 2010 BLS data on gender and age workforce composition by industry (Borberly J, personal communication) were combined with depression prevalence estimates from the National Comorbidity Study in employed individuals by gender and age [17] to estimate the number of full-time employees in the company expected to meet criteria for major depression each year.

Wage and Fringe – Year 2010 BLS industry category was used to generate 2009 BLS estimates for non-supervisory wage and fringe benefits for each of 14 industries.

### Productivity loss

#### Method 1: traditional compensation

Consistent with previous studies [7,18], the research team summed lost absenteeism work hours due to depression (hereafter referred to as absenteeism hours) and lost presenteeism work hours due to depression (hereafter referred to as presenteeism hours) over one year. Absenteeism hours were estimated by multiplying the number of depressed employees in each company (see Prevalence above) by annual absenteeism hours attributable to depression reported in a nationally representative sample of employees [18] adjusted for probability of health-related absenteeism in the industry [19]. A parallel process was used to calculate presenteeism hours.

#### Method 2: traditional compensation corrected for production disruption

Consistent with evidence that absent employees disrupt the company’s capacity to perform time-sensitive work [8,9], disruption corrected absenteeism hours were estimated by multiplying Method 1 absenteeism hours by an empirically-based industry-specific multiplier that estimates the impact of absenteeism on production disruption [19]. This multiplier was derived from a study asking 800 managers to estimate the impact of employee absenteeism on team performance by occupation. These occupation-specific estimates were then translated to industry-specific estimates by multiplying the disruption factor associated with each occupation by the distribution of occupations in the industry. The field faces methodological challenges in developing presenteeism multipliers for disruption correction. Previous studies note that 1/3 of managers report that the company absorbs loss from only a portion of presenteeism hours, a finding suggesting that managers observe coworker compensation for impaired on-the-job employees [20]. Rather than assume the overall cost of presenteeism has to be at least equivalent to the reduction in effective work hours as previous research has done [20], the research team made no adjustments to Method 1 presenteeism hours to correct for disruption.

#### Method 3: traditional compensation corrected for friction costs

Consistent with evidence that lost productivity in the workplace is often compensated for by others, [21-23], each respondent was asked to describe labor substitution in their workforce in response to one-week illness (see Table 2). The choice of a one-week period reflected our intent to understand labor substitution for moderate periods of depression absence (e.g., absences more than a ‘mental health day’ and less than short term disability). The study utilized respondent reports of labor substitution, noting that supervisor and employee reports on labor practices agree in 65% of cases in the one week time-frame we investigated [22]. To calculate friction corrected absenteeism, the research team estimated non-completed work hours as absenteeism hours minus substitution hours. Substitution hours were defined as the hours of work completed by temporary workers or coworkers/self at a later time. Temporary worker hours, estimated by multiplying absenteeism hours by the probability that a temporary would be hired, were subtracted from absenteeism hours to estimate partially non-completed hours. Coworker/self-substitution hours, estimated by multiplying partially non-completed work hours by the probability that coworkers/self would complete work, were then subtracted from partially non-completed work hours to estimate non-completed work hours. Friction corrected presenteeism hours were adjusted by multiplying presenteeism hours by the probability that the work assigned to a depressed employee would be completed by a coworker or the employee when she/he felt better, reflecting the lack of evidence that companies hire temporary workers to compensate for presenteeism [20].

**Table 2:** **Items used to assess labor practices**

**Construct**	**Item**
Sick leave	Please check the answer that best describes your company’s sick leave benefits: (a) paid sick leave, (b) paid sick leave as part of paid time off, or (c) no paid sick leave.
Probability of hiring temporaries when employees are absent	When an employee in your company *misses work because of illness for one week*, how likely is your company to hire a temporary worker? (a) never hire temporary workers, (b) seldom hire temporary workers, (c) sometimes hire temporary workers, (d) often hire temporary workers, or (e) always hire temporary workers^*^
Probability of work completion by coworker/employee when employees are absent	This next question asks you about employees in your company who are not replaced by temporary workers whey they are absent. When an employee in your company *misses work because of illness for one week*, how much of that employee’s work will be completed by coworkers or the employee him/herself upon return? (a) 0%, (b) 25%, (c) 50%, (d) 75%, or (e) 100%.
Probability of work completion by coworker/employee when employees are non-productive	When an employee in your company *attends work but is not productive for one week*, how much of that employee’s work will be completed by coworkers or the employee him/herself when the employee feels better? (a) 0%, (b) 25%, (c) 50%, (d) 75%, or (e) 100%.

^*^Scored as never =0%, seldom =25%, sometimes =50%, often =75%, always =100%.

### Cost of productivity loss

#### Method 1: traditional compensation

The sum of absenteeism and presenteeism hours was multiplied by the hourly wage plus fringe for the industry before dividing by the number of employees in the organization to estimate annual per capita productivity loss costs.

#### Method 2: traditional compensation corrected for production disruption

The sum of disruption corrected absenteeism hours and presenteeism hours was multiplied by the hourly wage plus fringe for the industry before dividing by the number of employees in the organization to estimate annual per capita disruption corrected productivity loss costs.

#### Method 3: traditional compensation corrected for friction costs

Friction corrected absenteeism costs were estimated as the sum of non-completed work costs, temporary worker costs and overtime costs [22,23]. Because employers did not report the proportion of lost work compensated by overtime paid to other employees, we allocated absenteeism compensated by overtime as follows. First, work hours completed by coworkers/self were equally divided among coworkers in regular time, coworkers in overtime, and self at a later date. In companies who provided sick leave, non-completed work hours were multiplied by 1.0, temporary worker hours by 1.25 [24], and overtime hours by 1.50 [25] of the average wage plus fringe for the industry. These choices reflected that companies pay base costs to the absent employee plus differential costs to the substitute [26]. In companies who did not provide sick leave, non-completed work hours were multiplied by 0, temporary worker hours multiplied by 0.25 and overtime hours by 0.50 of the average wage plus fringe for the industry. These choices reflected that companies pay only the differential cost of the substitute employee [27]. We assigned no dollar value to productivity loss for work completed by co-workers without overtime, assuming that co-workers maintain normal productive output despite greater strain for the short absence periods that depressed employees report. Friction corrected presenteeism costs were defined by multiplying friction corrected presenteeism hours by wage plus fringe for the industry. Friction corrected absenteeism and presenteeism costs were then summed before dividing by the number of employees in the company to estimate annual per capita friction corrected productivity loss costs.

### Data analysis

Respondents provided complete data on all items in this analysis except four labor practice questions, where missing data ranged from 6.5%-13.9% of respondents. Using multiple imputation [28], we calculated friction corrected estimates of productivity loss from these four labor practice questions for all respondents. Because productivity loss distributions for all three methods could not be normalized by standard transformations, we bootstrapped 1000 samples of 325 respondents in five imputed datasets before pooling results. For between method comparisons, the research team determined whether the three methods produced significantly different estimates by examining bootstrapped p values in the five imputed datasets [29]. We describe the level of agreement across the three estimates using a moment-method concordance correlation coefficient appropriate for non-normally distributed data [30,31] applied to the original sample. For within methods comparisons, we qualitatively examined the 10-90% confidence intervals around bootstrapped medians to characterize the average spread of loss estimates as a proportion of the median. We examined between and within method comparisons by size and by industry, restricting industry comparisons to the 5 industry categories which had 30 or more companies in the sample. All productivity loss costs are presented in 2009$.

## Results

The 325 participating companies representing a broad range of industries are described in detail in Table 3. Virtually all companies offer paid sick leave; however one third of companies offered paid sick leave through paid time off, where employees who do not use their sick leave can take the days as vacation days. Almost half of companies never hire temporary workers. The majority of companies report that half or more missed work is made up by coworkers or employees themselves at a later time when an employee is absent with a short-term illness.

**Table 3:** **Organizational characteristics (n = 325)+**

Number of physically distinct U.S. worksites (SD)	21.9 (107.3)
Size	
% small (100 to 500 employees)	33.5
% medium (501 to 2500 employees)	30.8
% large (2501 plus employees)	35.7
Type	
% for-profit	56.1
% not-for-profit	23.5
% public sector	20.4
Company age (SD)	74.8 (47.1)
Industry	
% Agriculture	0.6
% Construction	1.8
% Education/Health	12.3
% Finance	6.5
% Information	2.8
% Leisure/Hospitality	13.2
% Manufacturing	22.5
% Professional	13.5
% Public Administration	14.5
% Retail	2.5
% Transportation/Warehousing	6.5
% Utilities	1.5
% Wholesale	0.9
% Other	0.9
Labor monitoring	
% with any absenteeism monitoring	73.2
% with any productivity at work monitoring	56.1
Mean number of health plan carriers (SD)	2.2 (2.4)
Insurance risk	
% fully insured	24.1
% self-insured	46.2
% mixture of full and self-insured	9.7
% with Employee Assistance Program	80.5
Mean expected % increase in health premiums (SD)	7.7 (5.9)
Labor practices	
Sick leave benefits	
% paid sick leave	59.7
% paid sick leave as part of paid time off	34.0
% no sick leave	6.3
Likelihood of hiring temporary worker when employee misses work because of illness for one week	
0%	45.3
25%	42.3
50%	8.3
75%	3.3
100%	0.7
Likelihood that employee’s work will be completed by coworkers/employee upon return when employees misses work for one week because of illness	
0%	9.7
25%	12.5
50%	18.3
75%	33.9
100%	25.6
Likelihood that employee’s work will be completed by coworkers/employee at a later date when employees attends work but is not productive for one week	
0%	10.1
25%	21.5
50%	20.8
75%	15.8
100%	31.9

+Sample size varies from 280 to 325 due to missing data. Labor practices for missing items generated by multiple imputation. Percentages may not add to 100% because of rounding error.

Table 4 shows that the average company in the sample realized an annual $617 (SD = $75) per capita productivity loss as calculated by compensation methods, a $649 (SD = $78) loss as refined by disruption correction methods and a $316 (SD = $58) loss as refined by friction correction methods. In between method comparisons in the total sample, friction correction resulted in a proportionately larger absolute correction (50%) to the compensation method than disruption correction (105%). Friction correction means differed from compensation and disruption corrected means (p < .0001), but differences between compensation and disruption means were not consistently different from each other across the five imputed datasets. Reductions in friction-corrected presenteeism costs were qualitatively greater than reductions in friction-corrected absenteeism costs compared to compensation estimates. The concordance correlation coefficient (CCC) across all three estimates was 0.92 (95% CI 0.90, 0.93). Table 5 shows that similar between method differences were observed in analyses stratified by size and industry.

**Table 4:** **Annual per capita productivity loss estimates across three methods for total sample (n = 325)**

		**Compensation**	**Disruption correction**	**Friction correction**
**Productivity loss**				
Per capita productivity costs	Mean (SD)	617 (75)^*^	649 (78)^*^	316 (58)^*^
	Median (CI)^+^	611 (524 719)	643 (553 757)	309 (246 396)
	Ratio ^++^	NA	1.05	0.50
**Absenteeism loss**				
Per capita absenteeism days	Mean (SD)	3 (6)	3 (6)	1 (3)
	Median (CI)^+^	2 (1 3)	2 (1 3)	1 (0 2)
	Ratio^++^	NA	1.00	0.42
Per capita absenteeism costs	Mean (SD)	96 (210)	128 (277)	62 (152)
	Median (CI)^+^	71(26 109)	94 (36 154)	36 (3 101)
	Ratio^++^	NA	1.34	0.65
**Presenteeism loss**				
Per capita presenteeism days	Mean (SD)	16 (34)	16 (34)	8 (27)
	Median (CI)^+^	12 (7 18)	12 (7 18)	3 (0 12)
	Ratio^++^	NA	1.00	0.50
Per capita presenteeism costs	Mean (SD)	521 (1129)	521 (1129)	254 (917)
	Median (CI)^+^	389 (156 602)	389 (156 602)	122 (0 419)
	Ratio^++^	NA	1.00	0.49

SD = standard deviation, NA = not applicable, + CI =10^th^ and 90^th^ percentile confidence interval around median, Ratio^++^ = ratio of disruption correction mean (or friction correction mean) to compensation mean. ^*^Friction correction differs from compensation and disruption correction mean p < .0001.

**Table 5:** **Annual per capita productivity loss estimates across three methods by size and industry**

		**Compensation**	**Disruption correction**	**Friction correction**
**By size**				
Less than or equal to 10% (n = 34)	Mean (SD)	469 (25)	494 (26)	207 (27)
	Median (CI)^+^	470 (438 501)	494 (461 527)	207 (174 240)
	Ratio^++^	NA	1.05	0.44
Greater than 10% and less than 90% (n = 261)	Mean (SD)	658 (88)	692 (92)	343 (67)
	Median (CI)^+^	653 (549 782)	687 (578 822)	336 (259 436)
	Ratio^++^	NA	1.05	0.51
Greater than or equal to 90% (n = 30)	Mean (SD)	393 (47)	415 (50)	173 (39)
	Median (CI)^+^	396 (333 449)	418 (352 475)	172 (125 223)
	Ratio^++^	NA	1.06	0.44
**By industry**				
Education/Health (n = 40)	Mean (SD)	471 (47)	496 (49)	226 (39)
	Median (CI)^+^	466 (414 536)	490 (436 564)	223 (178 274)
	Ratio^++^	NA	1.05	0.48
Leisure/Hospitality (n = 43)	Mean (SD)	167 (13)	176 (14)	80 (11)
	Median (CI)^+^	167 (149 184)	176 (157 193)	79 (67 93)
	Ratio^++^	NA	1.05	0.47
Manufacturing (n = 73)	Mean (SD)	699 (120)	732 (125)	353 (86)
	Median (CI)^+^	704 (529 855)	739 (557 894)	353 (237 471)
	Ratio^++^	NA	1.05	0.50
Professional (n = 44)	Mean (SD)	643 (125)	672 (131)	257 (63)
	Median (CI)^+^	592 (526 869)	619 (550 908)	245 (209 291)
	Ratio^++^	NA	1.04	0.41
Public administration (n = 47)	Mean (SD)	687 (97)	727 (101)	391 (73)
	Median (CI)^+^	683 (554 821)	723 (588 869)	392 (290 492)
	Ratio^++^	NA	1.06	0.57

SD = standard deviation, NA = not applicable, CI^+^ = 10^th^ and 90^th^ percentile of median, Ratio^++^ = ratio of disruption correction mean (or friction correction mean) to compensation mean.

In within method comparisons in the total sample, the average 10^th^-90^th^ percentile spread of annual per capita lost productivity costs was 35.1% of the median average. In within method comparisons by size, the average spread for small companies was 16.6% of median average, 39.0% for midsize companies, and 34.0% for large companies. In within method comparisons by industry, the average spread was 22.6% of the median average for leisure/hospitality, 29.2% for education/health, 41.7% for public administration, 49.9% for manufacturing and 53.8% for professional.

## Discussion

In between method comparisons, this study found that the $617 per capita productivity loss derived from compensation methods increased 5% with disruption correction and decreased 50% with friction correction. The 50% decrease with friction correction is within the range of the 28-57% reduction reported in two Dutch studies [32,33]. Friction corrected estimates were statistically smaller than estimates produced by compensation methods and disruption corrected methods, which did not consistently differ from each other. In within method comparisons, the distribution of lost productivity estimates around the median qualitatively differed by company size and industry. Contrary to our hypothesis, results indicate a near perfect correlation of productivity loss across the three methods. These findings have implications for the intervention developers, business leaders, and health policy experts.

Intervention Developers – National working groups charged with identifying research priorities for the health promotion field highlight the importance of return on investment research and standardized methods for establishing these estimates [32,33]. This priority reflects that almost 50% of companies in the current sample note that return on investment was very significant in their choice of health benefits initiated in the past year [34]. In order to estimate a comprehensive estimate of return on investment, researchers are encouraged to evaluate programs for their impact on presenteeism as well as the more commonly studied indicator, absenteeism. Program impact on absenteeism may become increasingly less relevant as 52% of US companies in a 2013 national survey offered their workforce paid time off (providing paid days that employees can use for vacation or sick leave) rather than paid sick leave [35]. Intervention developers designing programs for employer purchase are encouraged to estimate the value of work productivity improvement from the compensation perspective and its two refinements to generate a range of return on investment estimates. Although it will be more difficult to demonstrate that an intervention has a return on investment using friction correction methods, it may be easier to disseminate the intervention using a conservative estimate of return on investment to employers who feel ‘burned’ by broken promises of return on investment for products they have previously purchased.

The high concordance coefficient indicates that the three methods are virtually interchangeable in economic analyses which do not rely on dollar value per se. For example, companies with high productivity losses identified by compensation costs are likely to be the same companies identified by friction costs. However, cost benefit evaluations [36-39] conducted from the employer perspective (cost of intervention compared to cost of productivity loss averted) may be biased by selection of method, with compensation and disruption corrections more likely to show a return on investment than friction methods.

Business Leaders - The substantial variation of lost productivity costs *between methods* underscores that method selection is an important issue for business leaders interested in achieving a positive return on investment on new health benefits. Friction correction, which accounts for labor substitution when employees are absent or impaired at work, estimates only *one-half the productivity loss costs* of the traditional method. These findings encourage business leaders in industries where labor substitution is common to ask for friction corrected estimates of return on investment when they consider purchasing a product.

Health Policy Experts - The substantial variation of lost productivity costs *within method* in this sample suggests that further research is needed before policy makers conclude that employers as a stakeholder bloc incur substantial loss from absenteeism and presenteeism attributable to depression. The substantial variation of lost productivity costs that these employers report encourages dissemination researchers to consider whether productivity-enhancing interventions can be more effectively disseminated by identifying ‘which employers benefit’ (the market perspective) rather than ‘whether employers will benefit’ (the societal perspective). The development of evidence-based calculators can assist in this undertaking (http://www.caremanagementfordepression.org/newCalc/page1.html).

Limitations on these observations should be considered. First, the productivity losses we estimate are not intended to definitively characterize productivity loss from depression in American companies. In terms of external validity, we did not study a representative sample of employers in this study but rather a national sample of companies interested in strategies that they can use to ensure their depressed employees get high quality treatment. Our estimates clearly need to be replicated in nationally representative databases. In terms of internal validity, we could not identify data sources for all necessary variables to produce company-level estimates of employer productivity loss from illness; however, the dataset we analyzed contains either the necessary variables or the variables needed to derive the estimates of interest utilizing published methods. This ‘best available’ information limits definitive conclusions about return on investment estimates but should not substantially bias comparisons across the three methods.

While all three methods are based on strong conceptualization, their operationalization is a work in progress. In disruption correction, the research methods which provide clear answers on how absenteeism disrupts team production, yield less than clear answers about how presenteeism disrupts team performance. Thus, the current study’s application of best available disruption correction methods result in a close overlap with compensation estimates, because presenteeism accounts for 81% of lost work productivity in depression [18]. In friction correction, although human resource professionals readily answer questions about compensatory labor practices in their organization, the field lacks a company-level instrument validated against observational data, a deficit recognized by leading proponents of this approach [40] and a simple way to present friction methods to business leaders. When both disruption and friction methodologists successfully meet these challenges, research teams will be able to develop a hybrid model defining core components of productivity loss from both frameworks.

## Conclusions

This is the first study in the literature to compare estimates from three recognized methods to estimate lost work productivity, to characterize the agreement among these methods, and to describe the potential consequences of method selection for the dissemination of interventions to employers. Intervention developers designing programs for employer purchase are encouraged to collect the necessary data to estimate the value of work productivity improvement from the compensation perspective and its two refinements to generate a range of return on investment estimates. Business leaders in industries where labor substitution is common are encouraged to seek friction corrected estimates of return on investment for products advertised as delivering a return on investment from improved productivity. Health policy analysts are encouraged to target the dissemination of productivity enhancing interventions to employers with high losses, rather than to all employers.

### Availability of supporting data

The dataset in which these analyses were conducted will be available at the Policy Service Research Data Center (http://psrdc.fmhi.usf.edu) in the Department of Mental Health Law and Policy at the College of Behavioral and Community Sciences at the University of South Florida through November 2019.
